# Prognostic implications of serum lipid metabolism over time during sepsis

**DOI:** 10.1186/2197-425X-3-S1-A226

**Published:** 2015-10-01

**Authors:** SH Lee, JM Lee, CY Kim, MS Park, BH Park, WJ Jung, SY Kim, EY Kim, JY Jung, YA Kang, YS Kim, SK Kim, J Chang, KS Chung

**Affiliations:** Severance Hospital, Yonsei University College of Medicine, Seoul, Korea, Republic of Korea; Korea University Anam Hospital, Korea University College of Medicine, Seoul, Korea, Republic of Korea

## Intr

Despite extensive research and an improved standard of care, sepsis remains a disorder with a high mortality rate. Sepsis is accompanied by severe metabolic alterations. However, there are controversies about changes in lipid profiles in sepsis patients.

## Objectives

To examine whether disorders of lipid metabolism are associated with worse disease activity and mortality.

## Methods

We evaluated 117 sepsis and septic shock patients (65 survivors and 52 non-survivors) who were admitted to the intensive care unit of a university-affiliated hospital in Korea. Serum levels of cholesterol, triglyceride (TG), high-density lipoprotein (HDL), low-density lipoprotein (LDL), free fatty acid (FFA), and apolipoprotein (apo) A-I were measured on days 0, 1, 3, and 7. Patients who had previously used statins or steroids, for treating lipid diseases or liver diseases were excluded.

## Results

Non-survivors showed significantly higher SOFA scores than survivors (p = 0.008). Non-survivors had low levels of cholesterol, TG, HDL, LDL, and apo A-I levels on days 0, 1, 3, and 7. In linear mixed model analysis, over time the variation in TG, LDL, FFA and apo A-I between groups differed significantly (p = 0.043, p = 0.020, p = 0.005, and p = 0.015, respectively). However, there were no significant differences in cholesterol or HDL between groups. Through multivariate analysis, TG level and SOFA scores were associated with mortality on day 0 and day 1 (p = 0.018 and p = 0.008, respectively). In survival analysis, the high TG level group showed better prognosis than the other groups (p = 0.022).

## Conclusions

Recently, in two large, multicenter, double-blind, randomized, placebo-controlled studies, simvastatin and rosuvastatin did not improve clinical outcomes in acute respiratory distress syndrome (ARDS) or sepsis-associated ARDS patients. Similary, in contrast to previous studies, our study showed that low TG level is associated with mortality in ICU patients with sepsis. This may be due to alterations in serum lipid metabolism during sepsis, modulating the host response to inflammation in critically ill patients.
